# Heads-Up: Risk-Specific Neurodegenerative Mortality and Years-Saved Analysis on the US Olympian Cohort

**DOI:** 10.3389/fphys.2021.705616

**Published:** 2021-09-09

**Authors:** Moi Yamazaki, Quentin De Larochelambert, Guillaume Sauliere, Jean-François Toussaint, Juliana Antero

**Affiliations:** ^1^Institute of Biomedical and Epidemiological Research in Sport (IRMES), National Institute of Sport, Expertise, and Performance (INSEP), Paris, France; ^2^Sorbonne Paris Cite, University Paris Descartes, Paris, France; ^3^CIMS, Hôtel-Dieu, AP-HP, Paris, France

**Keywords:** neurodegenerative mortality, sports-related traumatic brain injury, Olympians, longevity, survival

## Abstract

**Purpose:** This study aimed to identify the risk of neurodegenerative death (ND) that former Olympians endure due to their participation in sports grouped based on presumed repeated shocks to the head, and to understand the impact of their participation in such elite sports on their total longevity.

**Materials and Methods:** The cohort included all former US Olympians, who participated in the Olympic Games (OG) between 1948 and 1972, and whose vital status and causes of death were verified (*n* = 2,193). Olympic sports were classified into three categories of exposure: Collision (the highest presumed risk of repeated shocks to the head), Contact, and No-Contact. The Fine-Gray competing risk regression model was used to compare the risk of ND where the No-Contact category was a reference group. The years-saved analysis was performed to quantify the number of years saved or lost to ND and total longevity compared with the US general population.

**Results:** A total of 65 NDs were identified. Collision sports Olympians had a 3.11 (95% CI: 1.31–7.40) higher risk of ND while the Contact group showed a risk of 0.56 (95% CI: 0.21–1.48) compared with the No-Contact sports Olympians. Compared with the general population, the Collision group lost 0.61 (95% CI: -1.16—0.06) years of life from ND, while the Contact group saved 0.4 (95% CI: 0.26–0.54) and the No-Contact group saved 0.09 (-0.09–0.28) years of life up to the age of 90. Regarding the total longevity, Collision, Contact, and No-Contact groups saved 4.67 (95% CI: 3.13–6.22), 5.8 (95% CI: 4.93–6.67), and 6.24 (95% CI: 5.57–6.92) years of life, respectively, from all causes of death.

**Conclusion:** There is an elevated risk of ND among US Olympians, who engaged in sports with the highest presumed risk of repeated shocks to the head compared with those exposed to no such hazard. Such risk does not jeopardize the total longevity among Olympians in Collision sports.

## Introduction

Evidence suggests an association between the risk of the development of neurodegenerative disorders and repeated traumatic brain injury as a result of significant shocks on the head while playing sports (Peskind et al., 2013; Faden and Loane, 2015; Crane et al., 2016). It is understood that sports-related traumatic brain injury results in sustained neuroinflammation, acceleration of age-related neurodegeneration, and ultimately death (Peskind et al., 2013; Faden and Loane, 2015; Prien et al., 2018). The occurrence of sports-related traumatic brain injury is associated with the level of contact and repeated shock on the head which occur during practice and games (Daneshvar et al., 2011; Peskind et al., 2013; Prien et al., 2018). Sports with high collision incidence, such as American football, boxing, and rugby, have higher reported incident rates of sports-related traumatic brain injury than the sports, such as swimming and athletics, where athletes come in contact less frequently and with less impact on the head (Prien et al., 2018). Previous studies have found that the risk of neurodegenerative death (ND; deaths caused by degenerative nerve diseases such as Alzheimer’s disease and dementia) among American football players and Finnish boxers to be higher than that of the general population (Sahler and Greenwald, 2012; Kettunen et al., 2015; Simmons et al., 2017). However, such studies have set the general population as the control group when it would be more appropriate to compare with elite athletes to account for the cohort effect. Furthermore, the impact of ND on the total longevity of elite athletes remains inconclusive. So far, it is established that elite athletes tend to live 3–7 years longer than the general population mainly due to the lower risk of cancer and cardiovascular death (Lehman et al., 2012; Antero-Jacquemin et al., 2015; Radonić et al., 2017; Nguyen et al., 2019; Antero et al., 2020). However, a previous study based on former US Olympians did not show any survival advantage from nervous system disorders when compared with the general population (Zwiers et al., 2012; Radonić et al., 2017; Antero et al., 2020). Based on previous findings, we hypothesized that Olympians who were engaged in sports where players purposely and frequently hit or collide with each other and with inanimate objects may be subjected to a higher risk of ND, which could lead to a negative impact on their total longevity.

## Materials and Methods

### Study Design

This is a retrospective cohort study on the risk of NDs among the US elite athletes grouped, according to their participation in Olympic sports, into Collision, Contact, and No-Contact.

### Studied Population

Olympians were included in the cohort if they participated in a minimum of one Summer or Winter OG between 1948 and 1972. The entry was limited from 1948 due to the potentially less accurate data on specific causes of death before this period, and the upper limit was set to have enough follow-up time since the initial signs and symptoms of neurodegenerative disorders were found around the age of 65. A total of 2,261 athletes met the inclusion criterion; however, 68 (3%) Olympians without the identified cause of death were excluded, resulting in 2,193 athletes (404 women and 1789 men).

**FIGURE 1:**
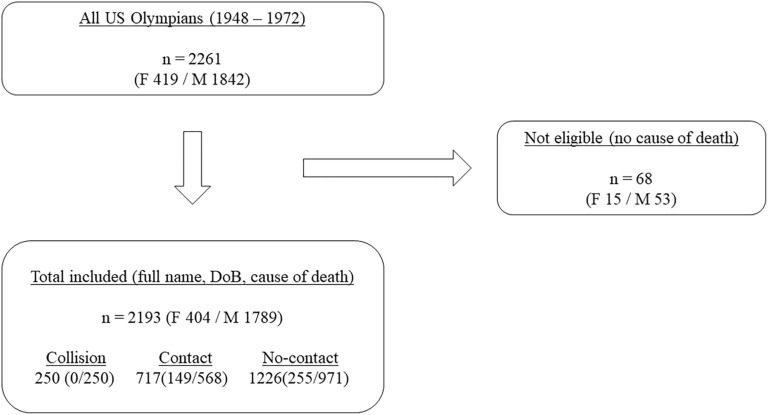
Flow diagram of cohort definition process (the 1948 cohort).

Although containing less accurate data on cause of death before the world wars, the cohort entry was extended to 1912–1972 and analyzed separately to have a greater number of subjects and be able to obtain a more generalized trend among the Olympians. The 1912–1972 cohort included 3,704 athletes, of which 19.6% (*n* = 726) of them were excluded due to missing data on the cause of death, resulting in a total of 2,978 athletes (492 women and 2,486 men) (Figure 1).

### Neurodegenerative Death

Deaths caused by degenerative nerve diseases are defined as NDs in this study (Lehman et al., 2012). The Olympians, who died from such diseases categorized under ICD9; Chapter 5; Section 290; Dementia and Chapter 6; Section 331–335; Alzheimer’s disease, Parkinson’s disease, Huntington’s disease, Friedreich’s ataxia, and motor neuron disease, were included (Lehman et al., 2012; Faden and Loane, 2015).

### Classification of the Risk

The categorization of sports was based on the presumed exposure to repeated shock on the head among players and with inanimate objects. Each sport was classified into one of the three categories, namely, Collision, Contact, and No-Contact (Table 1). The Collision category includes sports, such as boxing, ice hockey, judo, rugby, and wrestling, where players purposely and frequently hit or collide with each other and with inanimate objects (Zwiers et al., 2012; Reynolds et al., 2017; Mccradden and Cusimano, 2018; Čierna et al., 2019). In the Contact category, sports with less frequent and often not purposeful shock on the head (e.g., basketball, soccer, and gymnastics) were included (Table 1; Reynolds et al., 2017). The remaining sports were classified under the No-Contact category (e.g., athletics, archery, and swimming) (Ramkumar et al., 2019).

**TABLE 1:** Categorization of sports based on the risk of repetitive shock/jolt on head (Winter sports are italicized).

Collision	Contact	No contact
Boxing *Ice hockey* Judo Rugby Wrestling	*Alpen ski* Basketball *Biathlon* *Bobsleigh* *Cross country skiing* Cycling Diving Equestrian *Figure skating* *Freestyle skiing* Gymnastics Handball Hockey *Luge* Modern pentathlon *Nordic combined* Polo *Skeleton* *Ski jumping* *Short track speed skating* Soccer *Speed skating* Volleyball *Water polo*	Archery Athletics Canoeing Curling Fencing Rowing Sailing Shooting Swimming Tug-Of-War Weight lifting

### Data Collection

The list of athletes who represented the United States at the OG was obtained through a reliable online database^1^. This list includes the biographical information (e.g., full name, date of birth, place of birth, and sport participation) that were collected from official lists of Olympic competitors. Such data were available from 1912 until January 1, 2016, as the end point.

The vital status of each Olympian was confirmed through the Centers for Diseases Control and Prevention (CDC) using the National Death Index, which provides the underlying cause of death from 1979. Deaths occurred before 1979 and their underlying causes were identified through various reliable online resources. For the analysis, all the underlying causes of death were categorized and digitalized according to the International Statistical Classification of Diseases and Related Health Problems (ICD).

Berkeley Mortality Database provides the national life tables of the general population from 1900, while the Human Mortality Database contains the same information but from 1933. By combining these two, the overall mortality rate of the US general population was obtained. The mortality rate of neurodegenerative diseases of the general population was retrieved from the WONDER Database provided by the CDC. The data are available from 1968. Therefore, based on the trend from 1968 to 2016, the mortality rate of neurodegenerative disease of the general population was projected and used in this study.

The data collection process follows the existing study on the longevity of the US Olympians (Antero et al., 2020).

## Standard Protocol Approvals

This study received ethics approval from the Institutional Review Board at the University of Texas at Austin (2015-03-0035). Data analyses and protection have strictly adhered to the confidential data control plan based on the specifications set by the NDI.

### Data Availability

Biographical information of former US Olympians is publicly available; however, their causes of death confirmed through NDI^2^ are not open to the public.

The overall mortality of the US general population is available publicly through Berkeley Mortality Database and Human Mortality Database.

## Statistical Analysis

The baseline characteristics of the cohort were described using the mean (SD) or median (IQR) and proportions. The mortality risks and longevity were divided into two rounds of analysis. (1) The competing risk analysis using the Fine and Gray model, where the cumulative mortality rate of neurodegenerative disorders among Olympians is computed according to the risk of exposure. The competing risk model distinguishes the death caused by neurodegenerative disorders from non-NDs and prevents over/underestimation of the risk. In this analysis, Olympians in the No-Contact sports group were set as a reference, and the model included adjustment for the categories such as sex, medalist status, year of the first Olympic participation, and the body mass index (BMI), which followed the CDC definition (Division of Nutrition, Physical Activity, and Obesity, and National Center For Chronic Disease Prevention And Health Promotion., 2021); (2) The years-saved/lost analysis was performed to calculate the years saved if not lost from ND and all causes of death among Olympians compared with the general population. The second analysis enables us to quantify longevity losses or gains due to ND without neglecting the existence of competing risks for other causes of death. It maintains the rationality that the sum of years lost/saved by each cause corresponds to the total number of years lost/saved of life. First, we estimated the cumulative incidence function [F_k(t)] of the analyzed causes using the Aalen–Johansen method. This function was then compared with the one based on the general population of the same age and sex, living in the same calendar period obtained through the national life tables. Then, we calculated the difference between the areas below both functions to estimate the number of years lost or saved due to ND or all causes of death altogether (Antero et al., 2018; Duncombe et al., 2020).

In most, Olympic sports events, athletes compete at a young age (mean = 23.85 years old). However, in some sports (e.g., equestrian), athletes might participate on the Olympic level at an older age. While the number of such later-entry individuals is less, to diminish a potential age effect in the first OG participation, the beginning of the follow-up was conditioned at the age of 35 years. Additionally, to compare Olympians with the general population, the number of years saved was computed up to the age of 90 years. The final model included adjustment for sex and age. For both the competing risk model and the years-saved model, a visual investigation of 95% CI was done by checking the overlaps in the results from the 1948 to 1912 cohort to understand if the results from the 1948 cohort can be generalized when extending the cohort.

Statistical significance was determined based on 95% CI. All the analyses were completed by using R software (version 3.6.2).

## Results

In the 1948–1972 cohort, 33.6% (*n* = 737) had decreased by the end of the study period, and 65 (8.8%) deaths were caused by neurodegenerative disorders. The mean age of ND was 82.1 (*SD* = 7.74) years and the death in the oldest occurred at 96.7 years.

In the extended 1912–1972 cohort, 99 NDs were observed. The mean age of ND was at 84.1 years, and the death in the oldest occurred at 98.2 years. Other baseline characteristics are reported in Table 2.

**TABLE 2:** Description of the Olympians cohort characteristics.

Parameters	1948–1972			1912–1972		
		
	*N*	Female	Male	*N*	Female	Male
Neurodegenerative death (%)	65	4 (6)	61 (94)	99	11 (11)	88 (89)
ND among collision sports Olympians (%)	250	0 (0)	250 (100)	341	0 (0)	341 (100)
ND among contact sports Olympians (%)	717	149 (21)	568 (79)	930	177 (19)	753 (81)
ND among no contact sports Olympians (%)	1226	255 (21)	971 (79)	1707	315 (18)	1392 (82)
Mean age at the first OG in years (SD)	24 (5.16)	21 (4.41)	24 (4.98)	24 (5.05)	21 (4.41)	25 (4.87)
Mean age at leaving the cohort in years (SD)	74 (11.13)	71 (10.32)	74 (11.23)	76 (12.78)	73 (12.15)	76 (12.86)
Mean follow-up time in years (SD)	50 (10.75)	51 (9.46)	50 (10.97)	52 (12.78)	53 (11.66)	52 (12.97)

*Age in years, proportion, and IQR are rounded up.*

### The Risk of ND According to the Category of Sports

The cumulative mortality rates of the 1948 cohort for ND were 3.11 (95% CI: 1.31–7.40) among Collision sports Olympians (*n* = 250), and 0.56 (95% CI: 0.21–1.48) among Contact sports Olympians (*n* = 717) when compared with No-Contact Olympians (*n* = 1,226).

The results from the extended 1912 cohort are reported in Table 3 that shows convergent results with the 1948 cohort. The 95% CI from the extended cohort overlapped with the ones from the 1948 cohort.

**TABLE 3:** Results of cumulative mortality rate from neurodegenerative deaths among Olympians, adjusted (95% CI).

Parameters	1948–1972		1912–1972	
		
	Cumulative mortality rate	*P* value	Cumulative mortality rate	*P* value
Collision vs. No-contact	3.11 (1.31–7.40)	0.009	2.57 (1.11–5.94)	0.02
Contact vs. No-contact	0.56 (0.21–1.48)	0.25	0.73 (0.28–1.84)	0.50
Underweight vs. Normal	1.29 (0.09–18.15)	0.85	0.57 (0.06–5.26)	0.62
Overweight vs. Normal	1.67 (0.21–13.13)	0.63	0.92 (0.26–3.19)	0.90
Obese vs. Normal	1.77 (0.23–16.7)	*0.53*	1.08 (0.30–3.87)	*0.90*

### Neurodegenerative Mortality Among Olympians Compared With the General Population

**FIGURE 2:**
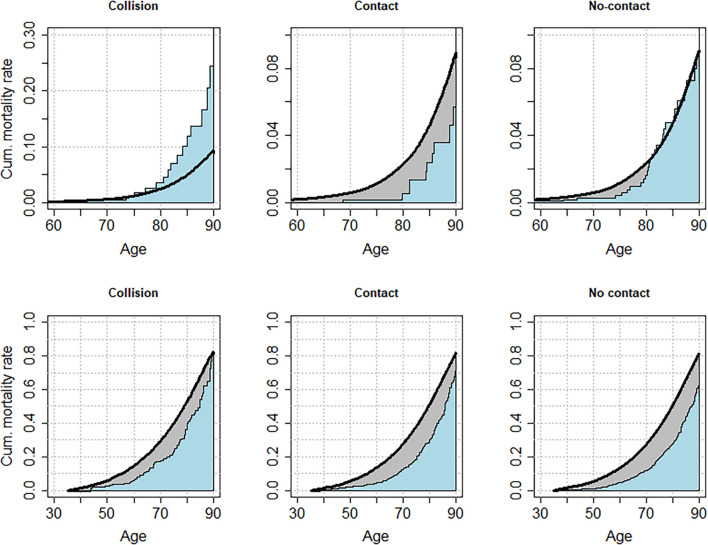
Cumulative mortality from neurodegenerative deaths (NDs) **(top row)** and all causes of death **(bottom row)** among US Olympians in the 1948–1972 cohort based on the participation in sports grouped according to presumed repeated shocks to the head. The black curve represents the expected cumulative mortality rate based on the mortality rates of the general population. The blue curve represents the cumulative mortality rate observed among Olympians. The shaded gray area represents the gap in favor of Olympians mortality rate compared with the general population.

**FIGURE 3:**
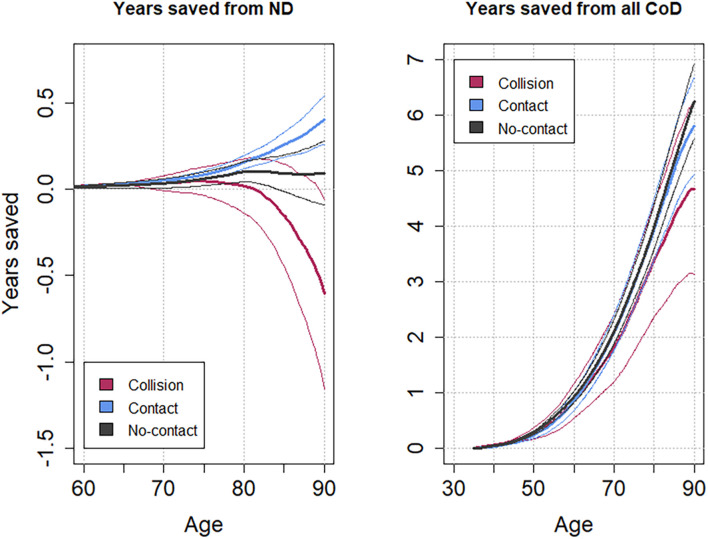
Years saved from NDs **(left)** and all causes of death **(right)** among US Olympians in the 1948–1972 cohort based on the participation in sports grouped according to presumed repeated shocks to the head. Thin curves represent each 95% CI limit.

Compared with the general population, the Collision group lost 0.61 (95% CI: -1.16—0.06) years of life from ND, while the Contact group saved 0.4 (95% CI: 0.26–0.54) years, and the No-Contact group saved 0.09 (95% CI: -0.09–0.28) year of life up to the age of 90 (Figures 2, 3).

Regarding the total longevity of Olympians, in the 1948 cohort, Collision sports Olympians saved 4.67 (95% CI: 3.13–6.22) years of life. Contact sports Olympians saved 5.8 (95% CI: 4.93–6.67) years, and No-Contact sports Olympians saved 6.24 (95% CI: 5.57–6.92) years of life from all causes of death (Figures 2, 3).

For the extended 1912 cohort, Collision sports Olympians lost 0.24 (95% CI: -0.53–0.06) years of life compared with the general population while both Contact and No-Contact Olympians saved 0.33 (95% CI: 0.23–0.42) years and 0.15 (95% CI: 0.04–0.25) years, respectively. Total longevity from the extended cohort showed a great survival advantage compared with the 1948 cohort; Collision and Contact sports Olympians saved 6.78 (95% CI: 5.51–8.05) years and 6.73 (95% CI: 6.0–7.46) years, respectively. No-Contact Olympians saved 7.06 (95% CI: 6.51–7.60) years.

## Discussion

### Main Findings

This study provides three major findings, namely, (1) the risk of NDs is more than three times high among the athletes who participate in the sports greatly associated with repeated shock on the head (where players purposely and frequently hit or collide with each other and with inanimate objects, e.g., boxing and wrestling) compared with the athletes who are not exposed to such risk; (2) Olympians in Collision group loses months of life due to increased risk of death from ND when compared with the general population. In contrast, Contact sports Olympians benefited from a survival advantage, since they gain months of life compared with the general population regarding the risk of ND; and (3) despite the elevated risk of ND, the US Olympians in the Collision group still experienced survival advantage considering all causes of death with prolonged longevity compared with the general population.

### Collision Sports

Previous studies have analyzed the risk of ND by comparing high-level athletes of one specific sport with the general population, which made it challenging to truly understand the risk associated with repeated shock on the head (Ljungqvist et al., 2009; Lehman et al., 2012; Kettunen et al., 2015; Nguyen et al., 2019). We found that Collision sports athletes, collectively, were 3.11 times at higher risk of ND when compared with No-Contact sports athletes. Even when including the Olympians from the extended cohort, a similar trend was observed. This suggests that sports with higher exposure to repeated shock on the head may be associated with a higher likelihood of ND. It also provides a generalized understanding of the risk in multiple sports regardless of its game style, highlighting that participation in Collision sports may expose athletes to a higher risk of ND, potentially affected by their occupation-specific hazards. However, this finding and its implication apply only to the male population. Due to the classification of sports, no woman participated in Collision sports in both the 1948 cohort and the extended cohort.

From a public health point of view, the observed trends may provide guidelines to protect elite athletes from potential ND. The International Olympic Committee (IOC) issued its first consensus statement on periodic health evaluation of elite athletes in March 2009, which included an assessment on concussion (a mild form of traumatic brain injury), yet did not put much emphasis on the importance of prevention (Mccrory et al., 2017). Indeed, when it comes to prevention of traumatic brain injury, especially concussion, protocols, and guidelines differ by sports and countries, often limited to educating coaches and athletes, and recommending and not mandating the use of protective gear such as a helmet. However, multiple studies have reported that the effect of protective gear and the reduction of risky practices (e.g., tackle) remain rather inconclusive, especially on the professional level (Kerr et al., 2015). Therefore, this study calls for clinical studies to provide specific prevention guidelines and protocols collectively on the sports with the highest risk of repeated head injury.

This study highlighted the risk endured by elite athletes presumably exposed to repeated shock on the head, which had been previously investigated only in few sports (e.g., American Football or boxing).

### Contact Sports

Contact sports Olympians from both 1912 and 1948 cohorts showed 30–40% decreased mortality when compared with No-Contact Olympians implying the lower risk of ND. There are a few possible explanations of why the decreased risk was observed in this particular population. First, since in this category, we included sports in which shocks on the head may happen, the athletes followed up may not have experienced repeated shock on the head. Second, because of this uncertainty, there is a possibility of misclassification of sports. After all, there were no such data available on the number of head injury by sports events. Therefore, all the sports were categorized solely based on the understanding of authors on the incidence of repeated traumatic brain injury, which was concluded from existing literature on the matter. Additionally, the degree of the risk may be different even within the category. Some athletes (e.g., Alpen ski and soccer) may be exposed more to the risk than other athletes (e.g., cyclists and gymnastics) although these sports fall in the same category. The rate of concussion among soccer players is higher than that of gymnasts with 20.22 and 0.26 per 1,000 athlete exposures, respectively (Ljungqvist et al., 2009; Popa-Wagner et al., 2020).

### Years Saved From NDs

A previous study on the longevity of US Olympians revealed that the cohort benefited the average of 5 years of gain in life, mainly driven by the years saved from cancer and CVD deaths (Antero et al., 2020). However, the study did not explore the potential survival advantage considering the isolated neurodegenerative disorders.

We showed that Collision sports athletes were losing years of life from ND (YS = −0.68) yet still saving years from all causes of death considered together. This implies that the impact of ND does not jeopardize their advantage on total longevity, suggesting that the risk related to ND is lesser than the risk of other major causes of death, which had a great impact on the total longevity of the studied cohort. The results from the extended 1912 cohort showed a similar trend to the 1948 cohort. The results and understanding align with the study on National Football League (NFL) players, where the standardized mortality ratio was found to be higher for ND yet lower for all causes of death, which indicates that ND is not a threat to total longevity on the population level (Lehman et al., 2012; Nguyen et al., 2019). While the study on NFL players is based on only 10 NDs, this study had 99 cases and considered multiple sports, showing better accuracy of the executed statistical model and its obtained results. Nonetheless, it is still limited given the low number of ND, and it is not possible to identify substantial evidence for the trivial effects.

## Limitation and Strengths

It is acknowledged that the exposure of interest in this study is a limitation, since (1) the intensity or the number of previous shocks on the head, (2) the potential medical care for the injuries, or (3) risk factors such as the lifestyles of athletes beyond their career were not taken into account. It is uncertain if the medical care was given to athletes and the type of treatment they received. If the injuries were treated appropriately, athletes may have not sustained symptoms that could lead to neuroinflammation which is a great risk factor for the development of neurodegenerative disorders. Since the illnesses are prominent among the older population, there is a long period of the window where athletes are exposed to various risks after their retirement such as a change in lifestyle. The diet that is high in sugar and fat, and alcohol and tobacco addiction are found to be the risk factors for neuroinflammation that could lead to neurodegenerative disorders (Popa-Wagner et al., 2020). This study could not control for such cofounders who could have a potential impact on the results, changing the observed trend of ND among the Olympians.

On the contrary to other studies among elite athletes, where the reference group was the general population, this study set athletes in No-Contact sports as the reference. Moreover, the results of the years-saved analysis regarding ND revealed the actual survival advantage of athletes, which was previously unexplored.

Further research should focus on a larger sample of female elite athletes to better understand the trend of the risk-specific neurodegenerative mortality rate among the female Olympians as well as compared with the general population.

## Conclusion

Elite athletes engaged in Collision sports with potentially high and frequent shock on the head had a higher risk of ND in contrast to athletes of comparable level but competing in sports with almost no such exposure. It also revealed that athletes at the highest risk do not benefit from a survival advantage beyond their career considering the risk of ND when compared with the US general population, adding a new value to the field of study on the longevity of elite athletes. Additionally, the total longevity of the Olympians was 4–7 years longer than the general population proving the survival advantage of elite athletes regardless of the risk of ND.

## Data Availability Statement

The data analyzed in this study is subject to the following licenses/restrictions: The dataset cannot be shared publically as it contains identifiable and confidential data. Requests to access these datasets should be directed to corresponding author.

## Ethics Statement

The studies involving human participants were reviewed and approved by Institutional Review Board at the University of Texas at Austin (2015-03-0035). Written informed consent from the participants’ legal guardian/next of kin was not required to participate in this study in accordance with the national legislation and the institutional requirements.

## Author Contributions

MY designed and conceptualized the study, analyzed the data, and wrote the manuscript for intellectual content. QD analyzed the data. GS supervised the data analysis. J-FT and JA designed and conceptualized the study and reviewed the manuscript for intellectual content. All authors contributed to the article and approved the submitted version.

## Conflict of Interest

The authors declare that the research was conducted in the absence of any commercial or financial relationships that could be construed as a potential conflict of interest.

## Publisher’s Note

All claims expressed in this article are solely those of the authors and do not necessarily represent those of their affiliated organizations, or those of the publisher, the editors and the reviewers. Any product that may be evaluated in this article, or claim that may be made by its manufacturer, is not guaranteed or endorsed by the publisher.

## Footnotes

1
https://www.olympic.org/athletes

2
https://www.cdc.gov/nchs/ndi/index.htm

## References

[B1] Antero J. Pohar-Perme M. Rey G. Toussaint J. F. Latouche A. (2018). The heart of the matter: years-saved from cardiovascular and cancer deaths in an elite athlete cohort with over a century of follow-up. *Eur. J. Epidemiol.* 33 531–543. 10.1007/s10654-018-0401-0 29730745

[B2] Antero J. Tanaka H. Larochelambert Q. D. Pohar-Perme M. Toussaint J. (2020). Female and male US Olympic athletes live 5 years longer than their general population counterparts: a study of 8124 former US Olympians. *Br. J. Sports Med.* 55 206–212. 10.1136/bjsports-2019-101696 32727712

[B3] Antero-Jacquemin J. Rey G. Marc A. Dor F. Haïda A. Marck A. (2015). Toussaint JF. mortality in female and male french olympians: a 1948-2013 cohort study. *Am. J. Sports Med.* 43 1505–1512. 25813868 10.1177/0363546515574691

[B4] Čierna D. Štefanovský M. Matejová L. Lystad R. P. (2019). Epidemiology of competition injuries in elite European judo Athletes: a prospective cohort study. *Clin. J. Sport Med.* 29 336–340. 31241538 10.1097/JSM.0000000000000526

[B5] Crane P. K. Gibbons L. E. Dams-O’Connor K. Trittschuh E. H. Leverenz J. Keene C. (2016). Association of traumatic brain injury with late-life neurodegenerative conditions and neuropathologic findings. *JAMA Neurol.* 73 1062–1069. 10.1001/jamaneurol.2016.1948 27400367 PMC5319642

[B6] Daneshvar D. H. Nowinski C. J. Mckee A. C. Cantu R. C. (2011). The epidemiology of sport-related concussion. *Clin. Sports Med.* 30 1–17, Vii. 21074078 10.1016/j.csm.2010.08.006 PMC2987636

[B7] Division of Nutrition, Physical Activity, and Obesity, and National Center For Chronic Disease Prevention And Health Promotion. (2021). *Defining Adult Overweight And Obesity: Overweight & Obesity : CDC (Online).* Available online at: Https://Www.Cdc.Gov/Obesity/Adult/Defining.Html (accessed September 25, 2020).

[B8] Duncombe S. L. Tanaka H. Larochelambert Q. D. Schipman J. Toussaint J. Antero J. (2020). High hopes: lower risk of death due to mental disorders and self-harm in a century-long US Olympian cohort compared with the general population. *Br. J. Sports Med.* 55 900–905. 10.1136/bjsports-2020-102624 33214139

[B9] Faden A. I. Loane D. J. (2015). Chronic neurodegeneration after traumatic brain injury: Alzheimer disease, chronic traumatic encephalopathy, or persistent neuroinflammation? *Neurotherapeutics* 12 143–150. 10.1007/s13311-014-0319-5 25421001 PMC4322076

[B10] Kerr Z. Y. Hayden R. Barr M. Klossner D. A. Dompier T. P. (2015). Epidemiology of national collegiate Athletic association women’s gymnastics injuries, 2009-2010 through 2013-2014. *J. Athl. Train.* 50 870–878. 10.4085/1062-6050-50.7.02 26196702 PMC4629945

[B11] Kettunen J. A. Kujala U. M. Kaprio J. Bäckmand H. Peltonen M. Eriksson J. (2015). All-Cause and disease-specific mortality among male, former elite Athletes: an average 50-year follow-up. *Br. J. Sports Med.* 49 893–897. 10.1136/bjsports-2013-093347 25183628

[B12] Lehman E. J. Hein M. J. Baron S. L. Gersic C. M. (2012). Neurodegenerative causes of death among retired national football league players. *Neurology* 79 1970–1974. 10.1212/wnl.0b013e31826daf50 22955124 PMC4098841

[B13] Ljungqvist A. Jenoure P. J. Engebretsen L. Alonso J. Bahr R. Clough A. (2009). The international Olympic committee (IOC) consensus statement on periodic health evaluation of elite Athletes, march 2009. *Clin. J. Sport Med.* 19 347–365. 10.1097/JSM.0b013e3181b7332c 19741306

[B14] Mccradden M. D. Cusimano M. D. (2018). Concussions in sledding sports and the unrecognized “Sled Head”: a systematic review. *Front. Neurol.* 9:772. 10.3389/fneur.2018.00772 30279676 PMC6153360

[B15] Mccrory P. Meeuwisse W. Dvoøák J. Aubry M. Bailes J. Broglio S. (2017). Consensus statement on concussion in sport-the 5th international conference on concussion in sport held in Berlin, October 2016. *Br. J. Sports Med.* 51 838–847. 28446457 10.1136/bjsports-2017-097699

[B16] Nguyen V. T. Zafonte R. D. Chen J. T. Kponee-Shovein K. Z. Paganoni S. Pascual-Leone A. (2019). Mortality among professional American-style football players and professional American baseball players. *JAMA Netw. Open* 2:E194223. 10.1001/jamanetworkopen.2019.4223 PMC6632140 31125098

[B17] Peskind E. R. Brody D. Cernak I. Mckee A. Ruff R. L. (2013). Military- And sports-related mild traumatic brain injury: clinical presentation, management, and long-term consequences. *J. Clin. Psychiatry* 74 180–188; Quiz 188. 23473351 10.4088/JCP.12011co1c PMC5904388

[B18] Popa-Wagner A. Dumitrascu D. I. Capitanescu B. Petcu E. B. Surugiu R. Fang W. H. (2020). Dietary habits, lifestyle factors and neurodegenerative diseases. *Neural Regen. Res.* 15 394–400. 10.4103/1673-5374.266045 31571647 PMC6921346

[B19] Prien A. Grafe A. Rössler R. Junge A. Verhagen E. (2018). Epidemiology of head injuries focusing on concussions in team contact sports: a systematic review. *Sports Med.* 48 953–969. 10.1007/s40279-017-0854-4 29349651

[B20] Radonić V. Kozmar D. Počanić D. Jerkić H. Bohaček I. Letilović T. (2017). Mortality and causes of death among Croatian male Olympic medalists. *Croat. Med. J.* 58 263–269. 10.3325/cmj.2017.58.263 28857519 PMC5577651

[B21] Ramkumar P. N. Navarro S. M. Haeberle H. S. Luu B. C. Jang A. Frangiamore S. J. (2019). Concussion in American versus European professional soccer: a decade-long comparative analysis of incidence, return to play, performance, and longevity. *Am. J. Sports Med.* 47 2287–2293. 10.1177/0363546519859542 31303010

[B22] Reynolds B. B. Patrie J. Henry E. J. Goodkin H. P. Broshek D. K. Wintermark M. (2017). Comparative analysis of head impact in contact and collision sports. *J. Neurotrauma* 34 38–49. 10.1089/neu.2015.4308 27541183 PMC5198110

[B23] Sahler C. S. Greenwald B. D. (2012). Traumatic brain injury in sports: a review. *Rehabil. Res. Pract.* 2012:659652. 10.1155/2012/659652 PMC3400421 22848836

[B24] Simmons M. M. Swedler D. I. Kerr Z. Y. (2017). Injury surveillance of head, neck, and facial injuries in collegiate ice hockey players, 2009-2010 through 2013-2014 academic years. *J. Athl. Train.* 52 776–784. 10.4085/1062-6050-52.4.03 28662349 PMC5561779

[B25] Zwiers R. Zantvoord F. W. A. Engelaer F. M. van Bodegom D. van der Ouderaa F. J. G. Westendorp R. G. J. (2012). Mortality in former olympic athletes: retrospective cohort analysis. *BMJ* 345 E7456. 10.1136/bmj.e7456 PMC3521875 23241269

